# Medical Society of the County of Albany

**Published:** 1873-06

**Authors:** F. C. Curtis

**Affiliations:** Secretary


ART. III.—*Medical Society of the County of Albany*, Semi-Monthly Meeting, May 14th, 1873.

Reported by F. C. CURTIS, M. D., Secretary.

In the absence of the President and Vice-President, Dr. Henry March was elected president *pro tem.*

Dr. James S. Bailey remarked, that one of the members of the Society, Dr. Peter P. Staats, had completed fifty years in the practice of medicine; he had been always an acceptable member and had done much toward elevating the profession of medicine in this City, and by his urbanity and courtesy had won and maintained the respect and esteem of every member of the society. He moved that, as it has been customary heretofore to celebrate the fiftieth anniversary of physicians engaged in the profession belonging to the society, a Committee be appointed to make arrangements for the occasion.

On account of the small number present, the motion was laid on the table until the next meeting.

A communication from the Clerk of the Common Council was read, stating that a resolution had been passed by that body, inviting the physicians of the city to give their opinion as to "Whether the water of the Hudson River is sufficiently pure and wholesome for a City water supply." The next meeting was set apart for a discussion of the question.

Dr. J. M. BIGELOW, read the following paper on Rheumatic Peritonitis:

Rheumatic Peritonitis is regarded by some as an hypothetical disorder, by most as extremely rare.

Niemeyer says "Peritonitis very rarely occurs in persons previously healthy, and its occurrence is due to catching cold or to some unknown atmospheric influence, it is then called rheumatic peritonitis." Rokitanskey insists that "spontaneous or rheumatic peritonitis exists," and attributes it "to the metastasis of anomalous exanthematous processes, or of rheumatic poison, which tends to exudation," but he fails to tell us of the nature of the morbid material or of its method of generation. Flint observes "that exposure to cold may originate peritonitis" and excepting by reference, says nothing of the probable origin of peritonitis, as due to poison in the blood. Habershon, from an analysis of five hundred cases of peritonitis explicitly asserts—that peritonitis is never idiopathic in its origin, but when not traumatic or due to perforation of the viscera, it is dependent on either an extension of disease from adjoining viscera or on certain blood changes, such as occur in albuminuria, pyæmia, rheumatism, &c. Todd also indirectly affirms this conclusion by stating that the poison of rheumatism is akin to that of pyæmia, as the originator of peritonitis. Tanner strengthens these statements by ascribing, as a cause of peritonitis, "the continuation of the blood by morbid poison." Rheumatism is now regarded, as the product of a morbid, intrinsic, alteration of the blood, by which lactic acid, a morbid poison, is produced, hence Tanner may by implication support Habershon's statements.

We also assert, that previous attacks of rheumatism in early life, from six to ten years, furnish a predisposing cause to the subsequent peritonitis of youth or of adult life: this may afford an explanation of the origin of an attack of peritonitis, otherwise inexplicable.

Again, we sometimes, though very seldom, meet with a family among whose members the slightest cold or digestive derangement provokes pains in the joints and muscles, and a general pyrexia: in a few days these symptoms disappear; suddenly co-incident with their cessation tympanites, abdominal pain, troublesome diarrhœa, "colicky" pains, appear, to be followed by a severe illness of several days duration, sometimes though very rarely by sudden deaths. In these last cases there would seem to be an hereditary predisposition to rheumatic fever, and to the election of the peritoneum as the especial object of its baneful attacks.

During the puerperal state, there sometimes appear symptoms of rheumatism, termed by the French puerperal acute rheumatism. Peritoneal inflammation, sometimes presents among the other symptoms, swollen joints, febrile action, lithic acid urine, etc. So may and does, rheumatic peritoneal inflammation occur in rheumatism not connected with the puerperal state. The rheumatic poison frequently attacks the heart, the pericardium, the pleura, serous membranes, the meninges of the brain, why, then, can it not invade the peritoneum, a serous membrane, and even more obnoxious to recovery, when attached.

Anatomical Characteristics.—We have only seen the autopsy of one case of so called rheumatic peritonitis. The lesions were those of diffuse peritonitis. The exudations were, however, sero-fibrinous and abundant; lymph was plentifully present and formed a thin coating over the hollow viscera and the parietal portion of the peritoneum. The peritoneal sac contained a fluid united with lymph, more or less abundant; small abscesses were also scattered throughout the intestines, which were found to contain a sero-purulent fluid, of a greenish cast. Cleared of exudation the peritoneum was brittle, softened and in spots deeply injected.

The adhesions between the intestines and other viscera were slight, easily separated, and very extensive, compassing the whole peritoneal cavity.

The urine was rather scanty of a deep saffron color; its specific gravity 1029; it exhibited traces of the urates, was also slightly albuminous, and excepting a slight effusion in the chest, there was no evidence of any previous disease of the pleura or lungs.

Clinical History.—Rheumatic peritonitis is of a gradual development. A chilly feeling prolonged, and general or severe chill, with subsequent pains in the joints or neck, a sense of general soreness "a pounded feeling," admonishes the subject that some sickness threatens. Slight fever, exacerbating towards evening is noticed, and a dark puffy appearance under the eyes, is apparent. This condition may last for two or three days, when there will be noticed a tendency to an intermittent "colicky" diarrhœa, flatulence, depression of spirits, a furred tongue, which in the morning may be rather dry and glossy, but towards evening becomes slightly moist, pallid and flabby round the edges. Anorexia is present, the chin at times becomes slightly rosy, the mouth pallid; the sleep is restless; the subject complains of "a bad cold, which has settled over the whole system," and of a pain in the abdomen, which pressure, cathartics or other remedies fail to relieve. The temperature at this time ranges from $102\frac{3}{4}$ F. evening, to $101\frac{1}{2}$ F. morning. The pulse from 100 to 129 per minute. A slightly heavy feeling in the top and back of the head is complained of and great prostration, disproportionate to the severity of the symptoms present is noticeable; or again the sufferer will be laboring under rather a mild attack of acute articular rheumatism; or thirdly, rheumatic fever in its most painful forms may plainly exhibit itself: in all these cases the unusual prostration, above mentioned enforces early attention.

This attack of apparently rheumatic fever, in its mild or more aggravated form, fails to yield to the usual measures, when, suddenly the articular pains cease, a sense of general chilliness is complained of, and abdominal pain and tenderness are markedly present: in a short time, symptoms of diffusive peritoneal difficulty clearly show themselves. The change of the attack is abrupt; the peritoneal disease is quickly declared, and is in most cases supplemental to the sudden disappearance of general physical disturbance. Continued pain is a very prominent symptom; it is generally diffusive, very rarely is it wanting, occasionally is it paroxysmal. Coughing, sneezing, deep inspiration aggravate the pain, the respirations are consequently shortened in length, increased in frequency.

Pressure, sudden change of position, likewise produce severe distress. The knees and thighs are generally flexed, though sometimes dorsal or lateral decubitus is tolerated and affords great relief.

Tympanites is always present to a considerable extent; Constipation due to paralysis of the muscular coat of the bowels by collateral œdema is the rule; diarrhœa is a not infrequent exception. Vomiting seldom occurs, the pulse is frequent and small, ranging from 120 to 135. The temperature fluctuates between 101½ F. to 103 F. or even reaches 106 F. The mind is generally quite clear. Muscular rigidity is frequently present. As the abdomen becomes distended with flatus, the intestines now literally blown up with gas, press up against the diaphragm, cause compression of the lower lobes of the lungs, excessive hyperæmia of the uncompressed portions and dyspnœa; this disturbance of the pulmonic circulation may extend to the venous system, and impart to the patient a cyanotic hue. "The anxiety of the patient is pitiful;" and the alternate appeal for relief and desperate despair indicate his intense bodily sufferings and mental anxiety. At last the mind becomes cloudy, the sufferer grows apathetic, delirious and soon comatose. The pulse becomes smaller and more frequent, the body is covered with a cold sweat, asthenia supervenes, and the patient succumbs to the malady. Should the subject linger for some time, the tympanites decrease, but never wholly disappears, the fever exacerbates, consumes the strength and very tissues;" the skin becomes dry and scaly, the muscles flabby and relaxed, the limbs swollen and œdematous, and death occurs in the 3d to 6th week by asthenia. Convalescence, if established, is tardy and lingering; it is attended by more or less "colicky pains," abdominal tenderness and asthenia. Three or four months may elapse before natural vigor and strength begin to return. Most of those attacked with peritonitis die of the disease, "not bcause this affection is particularly ill borne by the organism" says Niemeyer, "but because it depends on the severe blood disease," and he further observes "we frequently see the rheumatic peritonitis, which occurs in otherwise healthy persons, terminate in cure, providing the exciting causes be removed soon enough."

Flint states, that in uncomplicated cases the prospect of recovery is good under judicious and early treatment. It may destroy life in twenty-four hours or in fourteen days. The average duration, when convalescence or tardy development of the disorder does not take place, is five or six days. The significant symptoms of rheumatic peritonitis are the sudden cessation of general articular pain, and rheumatic symptoms, and the almost concurrent pain, tenderness and tympanites in the abdomen. Undue prostrations and laborious dyspnœa, indicate great danger. The mode of death is by asthenia, as Flint observes "a progressive increase of the frequency and feebleness of the pulse, progressively increasing prostration, coldness of the hands and feet, hiccough, etc.," presage an unfavorable advance in the disease. When these symptoms improve, and the tympanites and muscular rigidity diminish, we may entertain hopes of recovery, or at least of delay of fatal termination.

Diagnosis.—Peritonitis is readily diagnosed from all other disorders, after its development; as the tenderness on pressure, tympanites, "colicky pain," "belly ache," at once separate it from all other disorders. Rheumatic colic, lumbo-abdominal neuralgia, hysteria, mesenteric neuralgia, hyperæsthesia of the skin of the abdomen, may each simulate peritonitis, but a careful study and analysis of the symptoms, the amenity to treatment of the above disorders, and the stubborness with which peritoneal difficulty yields to remedies, will generally afford an easy discriminative diagnosis.

Treatment.—The treatment of rheumatic peritonitis consists mainly in rest, opium and permanganato of potassa, with a liberal use of nourishing beef broths, milk, and alcoholic stimulants, when there has been time to prescribe for the rheumatic disorder, prior to the peritoneal invasion, permanganato of potassa in half grain doses should be given every hour; the bowels should be opened by an enema; and opium given freely so as to relieve pain, subdue inflammation and secure rest. Gelseminum also has been recommended and by some said to be successful when given in the form of the fluid extract, three minims every hour. Quinine also may be employed with a view to its tonic effects. Externally leeches, rubefacients, or turpentine stupes, ought always to be applied; spongio-piline and hot water applications are well spoken of by authors. Besides these, Niemeyer says that cold compresses to the abdomen, exert a most beneficial effect, and he agrees fully "with those who consider the application of leeches to the abdomen, the use of cold compresses and the internal administration of opium, as the most effective treatment." Flint and Prof. A. Clark, each affirm that our main reliance in treatment is opium, given fully and freely. Cathartics, mercury, and blisters are alike condemned by most clinical observers and successful practitioners; although there are those who affirm that epispastics do frequently afford relief and that mercury does arrest or check the inflammation.

When an early dyspnœa and cyanosis admonish us of the imminence of fatal termination. "Venesection" as a temporary expedient may "as" Niemeyer observes "be resorted to," "for we know of no other remedy" he adds, "to fulfill this urgent indication."

To relieve vomiting, ice is best administered; to check the diarrhœa, opium is to be preferred to all other astringents.

When convalescence is established, cod liver oil and quinine should be liberally prescribed. If the exudation be great, iodide of potassium and acetate of potassa in small doses should, if borne, be ordered, in connection with the various preparations of calisaya bark. Above all, fresh air, "judicious exercises in the open air," nutritious diet, quietude of mind and body, should be strictly enjoined.

Cathartics should be given with great caution, and not for at least six weeks after recovery; enemata are to be preferred.

The tendency to frequent relapses should always be borne in mind Becoming fatigued, exposure to cold, inattention to diet, should be strictly forbidden. I will narrate the history of a few cases illustrative of this subject.

**M.** Bayth, 18, by occupation a shop girl, was attacked by acute articular rheumatism February 12th, 1873, and presented all the symptoms of this disorder. On the morning of the 19th, the articular pains suddenly subsided and abdominal tenderness, tympanitis, and the symptoms of rheumatic peritonitis developed themselves in the sequence mentioned in the paper. The undue prostration was early manifested, the fluctuating temperature from 101 F. to 105° F. plainly present. Permanganate of potassa in one-tenth grain doses, opium freely, and according to the disease, nutritious brothes, terpentine stupes, constituted the treatment. After an illness of seven weeks she recovered, but at the present time she has not by any means recalled her wonted vigor or strength.

J. H. Man, aged 21, a carpenter, was attacked Dec. 23d, 1872, with slight symptoms of rheumatic fever, complained of a "pounded feeling over the whole body," general soreness, had no articular pains, excepting in the urist. About one week afterwards was attacked with peritonitis. Under the treatment previously spoken of, he recovered, so as to resume occupation in March.

In addition to these I have the notes of two other cases which have recovered under the treatment recommended; also of two who died, one in four days, the other two weeks after the first symptoms of general peritoneal, rheumatic disorders developed themselves.*

* Papers were also read by Drs. Curtis and DeZouche which may be found on preceding pages.

